# Tertiary Inpatient Palliative Care within Region-Wide Services: A Retrospective Examination of Psychosocial and Medical Demographics at Admissions

**DOI:** 10.3390/cancers15235578

**Published:** 2023-11-25

**Authors:** Andrea Feldstain, Lauren Buote, Janet M. de Groot, Jennifer Hughes, Aynharan Sinnarajah

**Affiliations:** 1 Tom Baker Cancer Centre, Alberta Health Services, Calgary, AB T2N 4N2, Canada; lauren.buote@albertahealthservices.ca (L.B.); jdegroot@ucalgary.ca (J.M.d.G.); 2 Cumming School of Medicine, University of Calgary, Calgary, AB T2N 1N4, Canadaasinnarajah@lh.ca (A.S.); 3 Foothills Medical Centre, Alberta Health Services, Calgary, AB T2N 2T9, Canada; 4 Werklund School of Education, University of Calgary, Calgary, AB T2N 1N4, Canada; 5 Department of Medicine, Queen’s University, Kingston, ON K7L 3N6, Canada; 6 Department of Medicine, Lakeridge Health, Oshawa, ON L1G 2B9, Canada

**Keywords:** palliative care, cancer, symptom burden, quality of life, tertiary palliative care unit, end-of-life

## Abstract

**Simple Summary:**

This study examined patient-reported outcomes from a local tertiary palliative care unit (TPCU; locally named the IPCU) compared to published outcomes from other Canadian TPCUs. A retrospective file review revealed that compared to published Canadian TPCU data, the IPCU population was younger with more advanced cancer, the rate of hospital deaths was lower, and discharge to preferred locations was better. This service is well integrated in a variety of palliative care services within the health region, providing care at appropriate levels of need. We interpret that when enveloped in well-organized services within the region, a TPCU may be better able to prioritize patients with later-stage disease, facilitate the management of symptom crises earlier, and improve the rates of discharge to preferred locations.

**Abstract:**

Palliative care offers symptom relief and improved quality of life. Tertiary palliative care units (TPCUs) focus on complex suffering under the care of specialist palliative physicians and interdisciplinary teams. The Intensive Palliative Care Unit (IPCU) is a TPCU integrated in well-developed region-wide palliative services in Calgary, Canada. We compared the population accessing the IPCU to published data from other Canadian sites. Methods: A retrospective chart review was conducted using 8 sample months over a 2-year period. We gleaned psychosocial and medical demographics alongside the self-reported symptom burden on the Edmonton Symptom Assessment System. Descriptive statistics were calculated. Results: Adults (*n* = 117) with cancer admitted to the IPCU were 5–10 years younger, had later-stage cancer, and had higher discharges to preferred locations than other published Canadian TPCUs. Up to two months before admission, most commonly reported symptoms were consistent with the outpatient literature although with higher reported intensity. Discussion: With more advanced disease, younger age, and elevated symptom burden before admission, the IPCU still discharged patients to preferred locations at higher rates than other sites. This may be due to integration in the region’s organized palliative care services. Conclusion: With proper integration, a TPCU may be able to improve quality of life and reduce deaths in hospitals.

## 1. Introduction

Palliative care is a subspecialty of medicine that offers improved quality of life for patients and their families [1]. It began in 1967 as an end-of-life treatment to ease suffering for patients dying from cancer [2,3]. Since its inception, palliative care has evolved multiple times [4] and is now optimally integrated early for any cancer and other chronic diseases [5,6]. As exemplified in Hawley’s Bowtie model [6], medical and palliative services might collaborate or work alongside each other, optimizing both disease-modifying and quality-of-life treatments. Through the continuum of disease, treatment might be more focused on disease modifications earlier in the trajectory with quality-of-life interventions as a complement. Later in the trajectory, quality-of-life treatments might increasingly become the focus of care as the role of disease-modifying treatments lessens. This coordination aims to manage disease according to the patient’s goals, improve quality-of-life regardless of stage, provide additional support for patients and families throughout medical care, and optimize preparation for death at the same time as garnering hope.

Palliative care has the potential to improve quality of life at any disease stage and with cost savings to healthcare [7]. However, referrals to palliative care typically occur late in the disease trajectory. This is even true in countries like Canada, an international leader in the discipline and whose clinicians’ report an understanding of palliative care’s scope and comfort in referring. This disparity in knowledge, stated referral comfort, and practice might be attributable to various system- and provider-level factors [8,9,10,11,12].

Acute or tertiary palliative care units (“APCUs” or “TPCUs”, as used herein) are usually found in large urban centres in tertiary cancer-focused or large hospitals. They play a role in admitting patients who have highly complex symptom burden, directly under the care of specialist palliative physicians. It is often resourced well with a dedicated interdisciplinary team, including medical, rehabilitation, and psychosocial–spiritual clinicians. Interdisciplinary treatment plans aim to collaboratively address multidimensional sources of suffering with the goal of ensuring comfort and quality of life. TPCUs may also serve as hospices, supporting dying and death [13,14].

In Calgary, Canada, there is a TPCU situated within region-wide, integrated palliative care services. Such integration has been found to reduce rates of hospital deaths, improve successful discharges home, improve the likelihood of important end-of-life conversations, the likelihood of offered or received psychosocial–spiritual support, and increase retrospective reports of “excellent” care by the bereaved [15,16,17,18]. This TPCU purposefully focuses on acute symptom management, with the goal of discharge to preferred location, and does not serve as a hospice. Thus, it is called the Intensive Palliative Care Unit (IPCU). The IPCU may differ from other Canadian TPCUs in that:

- It functions as one of multiple palliative services within the same city. These include outpatient and inpatient hospital consult services, palliative home care, and hospice;
- There are seven residential hospices in Calgary that function collaboratively but independently from the IPCU. Patients with a prognosis of less than three months could transfer to hospice for comfort-level care outside of the acute hospital environment.

Referrals to IPCU are largely from other palliative services. The local population served almost exclusively has cancer as their primary diagnosis (98%) [19]. The treatment goal is usually to discharge home or to hospice, depending on patients’ preferred location. Only 1% of the overall population accessing palliative services uses the IPCU as their first palliative care service, indicating the highly specialized role of this service [8,19].

Considering the aforementioned qualities of the IPCU, it is possible that it is serving a different population than other TPCUs. We sought to (1) assess medical characteristics, psychosocial characteristics, and report the symptom burden of patients with cancer who are admitted to the IPCU; (2) understand the role that the IPCU plays in a fully integrated palliative program with different services meeting different patient needs; and (3) compare these to other Canadian TPCUs in the published literature to determine if there are differences in patient populations served. Understanding this may help identify who is accessing this care and could inform quality improvement and/or development needs for ourselves and other programs.

## 2. Materials and Methods

### 2.1. Participants

Convenience sampling was used to identify patients with cancer who were admitted to the IPCU in January, April, July, or October of 2015 or 2016. Authors AF and LB conducted retrospective chart reviews of cancer centre charts for patients admitted in these months. This timeframe was chosen to represent one month per season and to thereby reduce bias that different seasons might impose (e.g., holidays). Patients who had declined to complete the measures described below were excluded from analyses. Patients without cancer were excluded as these measures are not standard assessment tools outside of cancer care in Calgary. This study was approved by the local ethics board (Study ID HREBA.CC-16-0262).

### 2.2. Demographic and Medical Information

Demographic and medical information collected for this study were cancer type, cancer stage, goals of care designation, age, gender, marital status, date of admission, and date of death. We also gleaned whether or not their cancer care chart indicated that they were involved with a palliative care service prior to IPCU admission.

### 2.3. Symptom Burden

The Edmonton Symptom Assessment System (ESAS) is a validated self-report measure for patients in palliative care [20,21]. Patient ratings of symptom burden are assessed on nine visual analogue scales from 0 (no (symptom)) to 10 (worst possible (symptom)) [22]. Symptoms rated are pain, tiredness, nausea, depression, anxiety, drowsiness, appetite, well-being, and shortness of breath. At the time of data collection, the IPCU was in the process of establishing a process to collect regular ESAS measurements. Therefore, ESAS data from time of admission to the IPCU were not available for our chart review. Instead, we collected the most recent ESAS information from cancer care charts just prior to IPCU admission.

### 2.4. The Canadian Problem Checklist

The Canadian Problem Checklist was developed to provide context to the ESAS item ratings [20]. Different centres can customize their list of problems based on common concerns their patient populations face. The local version used in 2015/16 had 48 items grouped into 6 categories: emotional, physical, social, informational, spiritual, and practical [22].

### 2.5. Data Analysis

Multiple imputation was conducted for missing values using NORM software [23]. This approach uses available data and variability in the existing dataset to estimate missing data points. It is a preferred method of data imputation in clinical research [24]. It was chosen given our exploratory methodology and minimal missing datapoints. Each of the nine ESAS symptom severity items were included for estimation. We also included a previous ESAS measure from the patient’s chart, if available, to bolster available data and thereby improve estimations. There were no a priori hypotheses in this exploratory study, and statistical analyses were conducted using SPSS Version 25 (IBM Corporation, Armonk, NY, USA). Descriptive statistics were used to describe the sample.

## 3. Results

A total sample of 138 participants with cancer were admitted to the IPCU during this 8-month sample period. There were five IPCU patients without a file in the cancer centre’s charting system, and we were therefore unable to confirm their diagnosis or acquire ESAS measurements. From the 138 patients confirmed to have cancer, we excluded 5 (3.6%) who did not complete the last ESAS before their admission (i.e., the questionnaire was left blank or chart indicated a refusal to complete) and 16 (11.6%) who did not have any ESAS questionnaires on file (e.g., new diagnosis, no previous visits to the cancer centre). Our final sample included 117 participants (84.8%) who had completed an ESAS before their admission. Missing values were replaced for 0.003% of the total dataset using multiple imputation.

Participants were 64 (54.7%) men and 53 (45.3%) women (See Table 1). The mean age was 60.3 ± 13.7, ranging from 22 to 85 years. The majority of participants were married (64.1% total). At the time of admission, 94.0% had stage IV disease. Primary tumour sites were lung (27.4%), gastrointestinal (26.5%), and breast (12.0%). Most patients (67.5%) had some specialist palliative service involvement prior to admission documented in their chart. Most were admitted close to death: 54.3% died within one month, 75.9% died within 3 months. Participants survived a median of 27.5 days (mean = 77.8, SD = 135) after admission, 24.8% died during their admission, and 12.9% survived 6+ months post-admission. See comparison to other published Canadian TPCU demographics in Table 1.

The ESAS was completed within the week before admission by 20.5% of the sample, within 1 month prior to admission by 58.1%, and within 2 months prior to admission by 79.5%. The highest rated symptoms were fatigue (mean = 5.8 ± 2.8, median = 6), pain (5.0 ± 3.0, 5), overall well-being (4.8 ± 2.5, 5), and drowsiness (4.5 ± 2.8, 4). The mean and median for these variables were all in the “moderate burden” category. Additional symptoms were scored in the “mild burden” range: appetite (3.9 ± 3.3, 3), shortness of breath (3.6 ± 3.1, 3), depression (3.2 ± 2.9, 3), anxiety (3.2 ± 2.9, 3), and nausea (2.3 ± 2.6, 1; see Table 2). Of the top 10 concerns reported on the Problem Checklist, 6 of these were in the physical category, 3 in emotional category, and 1 in the social category (see Table 3). The top three concerns were walking/mobility, sleep, and weight.

## 4. Discussion

In this study, we examined individual-level factors (medical, psychosocial, and subjective suffering) of the population being admitted to our local tertiary palliative care unit, the IPCU. We also compared these data to other published Canadian TPCU data. Compared with other TPCUs in Canada [18,25,26], the IPCU patient population was approximately 5–10 years younger and had almost exclusively advanced cancer. The sex distribution was fairly evenly split across all of these reports (49% to 54% women). The most common tumour sites (lung, gastrointestinal, breast, and prostate) were consistent with the top prevalences in Canada [27]. The IPCU death rate, 24.8% during admission, is lower than other published Canadian data (contrast 39.8–79.5%) [18,25,26]. It is also lower than the overall hospital mortality rate in Calgary for patients with cancer (41%) [8]. Internationally, the hospital mortality rate varies greatly (3.8–87%), possibly attributable to differing practices of early palliative referrals [14,15,16,17,18]. The rate of discharge home in our sample was 49.6%, including those in the final weeks or months of life, which is comparatively higher than other Canadian TPCUs (contrast 9.2–39%) [18,25,26]. Again internationally, this varies greatly with rates ranging from 10 to 91.2% [14]. Presumably, this is related to the availability of services and patterns of early/late palliative referrals (e.g., TPCU and hospice combined) [14,15,16,17,18]. These national and international comparisons suggest that Calgary’s IPCU meets its goal of acute symptom management with discharge and does not serve multiple roles like some TPCUs nationally and internationally.

Consistent with international findings on symptom burden at TPCU admission, the highest rated symptoms in our data were fatigue, pain, overall well-being, and drowsiness [28,29,30,31,32]. Sources of suffering were mostly physical (mobility, sleep, weight, performing activities of daily living, constipation, and cognitive concerns) and emotional (sadness, frustration, and fear), similar to provincial findings [33]. We compared categorical symptom burden (ESAS scores mild 0–3; moderate 4–6; severe 7–10) to published data from two other samples (see Table 2). One was outpatients with cancer receiving palliative care through an outpatient clinic at our centre [34]. We found that a higher proportion of our sample (47.9%) reported severe fatigue than local outpatients (33.3%) and less severe anxiety (13.7%) than local outpatients (17.6). Second, we compared the IPCU population to province-wide outpatients with cancer within 6 months of death [33]. We found that moderate-to-severe pain was endorsed by more of our patients (68.4%) than the contrast group (49.1%). Moderate-to-severe anxiety was endorsed more frequently (46%) by our sample (contrast group: 36%). These differences are consistent with the purpose of Calgary’s IPCU: to acutely manage high symptom burden.

Further integrating all findings, our results suggest that despite higher symptom burden with further progressed disease during admission, IPCU patients are still discharged at higher rates than other published TPCUs in Canada and some international rates. For the IPCU population, this may be attributed to multiple factors:

1. Patients can receive symptom management at any point in their illness trajectory: mild or moderate burden can be well managed on an outpatient basis, matching the right level of care for patient needs at that time. This can be offered to patients as early as diagnosis, if needed.
2. Already receiving outpatient palliative services would facilitate earlier admission for intensive palliation if/when symptom burden escalates beyond outpatient capabilities.
3. The availability of seven local hospices provides a destination when intensive palliation is no longer consistent with goals or needs, making IPCU beds available for other patients with acute symptom management needs.
4. In addition to the integration of palliative services, there is also local integration in other medical services, such as home care, other acute care medical centres, and community outreach. This integration improves the knowledge of available palliative services for a wider network of providers, bolstering the likelihood of patients in need being properly identified and referred before crisis.

### 4.1. Limitations

The reported data were collected for clinical purposes to enhance communication between patients and healthcare providers during routine outpatient visits. The clinical context compared to an evaluation context might affect how patients complete these questionnaires. Additional data that could be helpful for a program evaluation were also not necessarily available. At the time of data collection, the IPCU had not standardized distress screening and therefore measures at admission were not available. To account for this, we utilized what was available in cancer care charts, which yielded a time delay between measurement and admission. This data collection method of cancer chart reviews did not fully capture prior specialist palliative care involvement, as evidenced by the lack of referrals by the hospital palliative consult team, a primary referral source for the IPCU. Sociodemographic information was limited, and some variables were not routinely collected, such as identified gender or sexual identity, ethnicity, quality of social support, or socioeconomic status.

The data herein represent outcomes from an integrated palliative care network of services, in a high-income country. Our medical system is funded by both local and federal governments and also by charitable organizations. If the aforementioned hypotheses about mechanisms of action are correct, for other regions to obtain similar outcomes, the development of diverse and integrated palliative services would be needed. Funding sources would affect resources, limitations, and outcomes. This program development may be more realistic in regions of high-income countries that have existing palliative services whose palliative service network could be further developed and integrated. In medium or low-income countries, or in high-income countries with little palliative infrastructure, provision of basic palliative care may be more topical. Calgary, Alberta, is one major city in Canada. It differs culturally in terms of populations served and health care structure, among other diversity factors. There are likely inequities in who accesses health and/or palliative care services [35,36]. Our results may not be generalizable to other regions, including other Canadian cities.

### 4.2. Future Directions

Future directions include evaluating IPCU access pathways to better understand the differences in our findings versus other Canadian TPCU programs. Additional research should also be conducted on identifying and repairing health disparities in access to the IPCU and/or other palliative care services. This would require prospective data collection and a larger sample size. Screening for current, historical, or compounded trauma may help inform suffering, intervention, and potential outcomes. With this information, we may be able to identify gaps and barriers, to improve pathways, and inform the development of future programs.

## 5. Conclusions

When integrated in a system of palliative care services across a region, a TPCU may be better able to 1—focus on patients with later stage disease, 2—focus on managing moderate and severe symptom burden, and 3—reduce rates of death in hospital, bolstering the frequency of survival and death in preferred locations. With better symptom management and discharge from hospitals, patients and families may find better quality of life within their locations of choice, with more control in their environments, and without the chaos of an acute medical centre in their final months, days, or moments of life. Further research is needed to replicate these findings and examine factors in the IPCU that bolster these rates compared to other published Canadian TPCU data.

## Figures and Tables

**Table 1:** Demographics and medical information.

Characteristics, Patients (*n* = 117)	IPCU	Canadian TPCUs ^†^
Age		
Mean [SD]	60.3 [13.6]	64–75
Min-Max	22–85	
Sex *	%	%
Men	54.7	46–51
Women	45.3	49–54
Marital Status		
Married/Common Law	67.5	
Single/No Spouse Documented	16.2	
Divorced/Separated	11.1	
Widowed	5.1	
Survival (IPCU Admission to Death)		
Median	27.5 days	
Mean [SD]	77.8 [135.0] days	
1 month [0–4 weeks]	53.8%	
2 months [5–8 weeks]	12	
3 months [9–12 weeks]	9.4	
4 months [13–16 weeks]	6	
5 months [17–20 weeks]	5.1	
6+ months [21+ weeks]	13.7	
Diagnosis		
Cancer	96.5	32.6–40
Unknown or unspecified	3.5	
Cancer Stage at Admission		
I	0	
II	0.9	
III	5.1	
IV	94	
Previous Specialist Palliative Care		
Outpatient Palliative Care	35.9	
Palliative Homecare	29.9	
Other	1.8	
None indicated	32.5	
Primary Tumour Site		
Lung	27.4	
Gastrointestinal	26.5	
Breast	12	
Prostate	10.3	
Gynecological	7.7	
Head and Neck	4.3	
Neurological	4.3	
Other	7.8	
Discharged To		
Home	49.6	9.2–39
Another Facility	25.6	
Deceased	24.8	39.8–79.5

* This refers to assigned sex at birth, as reported in medical charts. This is not identified gender, which was not indicated in any of the charts reviewed. ^†^ Data gleaned from [18,25] and the most recent year reported in [26]. Not all variables were available in each of these reports.

**Table 2:** Symptom burden ratings on the ESAS.

Symptom (*n* = 117)	Mean (SD)	Median	Rates (%) of Endorsed ESAS Items ≤2 Months before IPCU Admission		
			Severe (7–10)	Moderate (4–6)	Mild (0–3)
Fatigue	5.8 (2.8)	6	47.9	27.4	24.8
Pain	5.0 (3.0)	5	32.5	35.9	31.6
Overall Well-being	4.8 (2.5)	5	23.9	47.9	28.2
Drowsiness	4.5 (2.8)	4	30.8	29.9	39.3
Appetite	3.9 (3.3)	3	28.2	21.4	50.4
Shortness of breath	3.6 (3.1)	3	19.7	29.1	51.3
Depression	3.2 (2.9)	3	16.2	27.4	56.4
Anxiety	3.2 (2.9)	3	13.7	32.5	53.8
Nausea	2.3 (2.6)	1	11.1	16.2	72.6

**Table 3:** Highest endorsed checklist problems.

Category (*n* = 117)	Concern	Percent
Physical	Walking/Mobility	51.3
Physical	Sleep	35.9
Physical	Weight	32.5
Physical	ADLs	30.8
Emotional	Sadness	29.9
Emotional	Frustration/Anger	29.9
Emotional	Fear	29.1
Physical	Constipation	26.5
Physical	Concentration/Memory	25.6
Social	Burden to Others	22.2

## Data Availability

The data are not publicly available due to the clinical nature of collection. With the information used for this study, patients are at risk of being identified.
